# Risk factors for depression in systemic lupus erythematosus: a systematic review and meta-analysis

**DOI:** 10.3389/fmed.2026.1751870

**Published:** 2026-02-19

**Authors:** Linling Zhu, Jinju Chen, Yaping Sha, Huiren Zhuang, Chunhua Ye

**Affiliations:** 1 School of Medicine, Tongji University, Shanghai, China; 2 Department of Nursing, Renji Hospital, Shanghai Jiao Tong University School of Medicine, Shanghai, China; 3 Shanghai East Hospital, School of Medicine, Tongji University, Shanghai, China

**Keywords:** depression, glucococorticoids, meta-analysis, risk factors, socioeconomic factors, systemic lupus erythematosus

## Abstract

**Background:**

Depression is highly prevalent among patients with systemic lupus erythematosus (SLE) and is associated with adverse clinical outcomes. However, evidence on its risk factors remains inconsistent, limiting early identification and targeted intervention.

**Methods:**

We conducted a systematic review and meta-analysis in accordance with PRISMA and MOOSE guidelines. Eight databases were searched from inception to May 2024 for observational studies reporting risk factors for depression in adult SLE patients assessed by validated scales. Study quality was evaluated using the Newcastle–Ottawa Scale or AHRQ criteria. Pooled odds ratios (ORs) with 95% confidence intervals (CIs) were calculated using random-effects models.

**Results:**

A total of 26 studies (*n* = 8,960 patients) were included. Significant risk factors for depression included economic hardship (OR = 6.05, 95% CI: 3.64–10.07), high-dose glucocorticoid use (OR = 7.72, 95% CI: 4.62–12.90), higher disease activity (OR = 3.15, 95% CI: 2.95–3.37), unemployment (OR = 3.06, 95% CI: 1.48–6.32), lower academic qualifications (OR = 2.21, 95% CI: 1.45–3.36), presence of comorbidities (OR = 2.20, 95% CI: 1.45–3.34), smoking (OR = 3.17, 95% CI: 1.44–6.99), and greater fatigue severity (per unit increase: OR = 1.23, 95% CI: 1.08–1.40). Younger age was also associated with higher depression risk (OR = 1.97, 95% CI: 1.41–2.76). Subgroup and meta-regression analyses revealed substantial heterogeneity across studies, partially explained by geographic region and depression assessment tools. Publication bias was detected but did not alter core findings in sensitivity analyses.

**Conclusion:**

This meta-analysis identifies key clinical, demographic, and psychosocial risk factors for depression in SLE. Findings support integrating routine depression screening with holistic assessments of disease burden and social context in clinical practice, particularly within nursing-led care models.

**Systematic review registration:**

https://www.crd.york.ac.uk/prospero/display_record.php?ID=CRD42024557892, identifier CRD42024557892.

## Highlights

- Identified key modifiable and non-modifiable risk factors for depression in systemic lupus erythematosus (SLE), including high disease activity, pain severity, low social support, female sex, and unemployment.
- Demonstrated substantial heterogeneity across studies, partially attributable to geographic region and depression assessment tools, highlighting the need for standardized outcome measurement.
- Provided evidence-based guidance for integrating routine depression screening with holistic psychosocial assessment in SLE clinical care, particularly within nursing practice.
- Confirmed the robustness of pooled estimates through rigorous sensitivity and publication bias analyses, supporting the reliability of the findings.

## Introduction

Systemic lupus erythematosus (SLE) is a chronic, multisystem autoimmune disease characterized by immune dysregulation, persistent inflammation, and autoantibody production–most notably antinuclear antibodies (ANA)–which can result in progressive organ damage (1). The condition disproportionately affects women, particularly during reproductive years (2). In mainland China, prevalence estimates range from 30 to 70 per 100,000 individuals (3), consistent with broader patterns across Asia (4).

While long-term survival has improved dramatically–with 5-years survival now exceeding 90% in many regions (5) –patients continue to face significant physical and psychological challenges due to the disease’s unpredictable course and lifelong management demands (6).

Depression is among the most common psychiatric comorbidities in SLE, affecting an estimated 24%–39% of patients (7), a rate substantially higher than in the general population or those with other rheumatic diseases (8). Critically, depression in SLE is not merely a reactive state; it independently predicts adverse outcomes, including cardiovascular events (9), suicidal behavior (10), functional disability (11), reduced quality of life (12), and increased mortality (13).

Despite growing recognition of its clinical significance, evidence on risk factors for depression in SLE remains inconsistent (14–18). Many studies focus narrowly on isolated clinical or demographic variables–such as age, disease duration, or renal involvement–while overlooking modifiable psychosocial determinants, including social support, illness perception, treatment burden, and health-related quality of life. This fragmented understanding hinders the development of effective screening tools and targeted interventions.

To address this gap, we conducted a systematic review and meta-analysis to: (1) identify robust risk factors for depression in adult SLE patients; (2) quantify the magnitude of their associations using pooled effect estimates; and (3) translate findings into actionable insights for clinical nursing practice. By integrating both biological and contextual predictors, our work aims to support early identification, multidisciplinary care coordination, and the design of patient-centered psychosocial strategies in real-world settings.

## Materials and methods

This meta-analysis adheres to the Preferred Reporting Items for Systematic Reviews and Meta-Analyses (PRISMA) statement (19) (See Supplementary Table 5) and the Meta-analysis Of Observational Studies in Epidemiology (MOOSE) guidelines (20). The protocol for this systematic review was prospectively registered in the International Prospective Register of Systematic Reviews (PROSPERO) before data extraction and analysis commenced, under registration number CRD42024557892.

### Research strategy

This meta-analysis was designed to identify risk factors associated with depression among individuals diagnosed with systemic lupus erythematosus (SLE). A comprehensive literature search was performed across eight electronic databases–Web of Science (WOS), the Cochrane Library, PubMed, Embase, China National Knowledge Infrastructure (CNKI), Wanfang Data, VIP Database, and Chinese Biomedical Literature Database (CBM)–with coverage up to May 2024. The search strategy combined Medical Subject Headings (MeSH) terms and relevant free-text keywords, including variations of “systemic lupus erythematosus” (e.g., “Lupus Erythematosus, Systemic,” “Disseminated Lupus Erythematosus”), along with terms related to depression (“depressive disorder,” “depression”) and risk assessment (“risk factors,” “risk indicators,” “health-related correlates”). An exemplar search string for PubMed is provided in Table 1. To further enhance retrieval completeness, reference lists of eligible and relevant publications were manually screened.

**TABLE 1:** Search methods.

Search strategy	Search terms
#1	(((SLE) OR (Systemic Lupus Erythematosus)) OR (Lupus Erythematosus Disseminatus)) AND (lupus erythematosus, systemic[MeSH Terms])
#2	(((depression[MeSH Terms]) AND (Depressive Symptoms)) OR (Depressive Symptom)) OR (Emotional Depression)
#3	(((risk factors[MeSH Terms]) AND (Risk Score*)) OR (Health Correlates)) OR (Population* at Risk)
#4	#1 AND #2 AND #3

The asterisk (*) functions as a truncation symbol in database search syntax, matching zero or more characters to retrieve all morphological variants of the root term (e.g., “Population” captures “Population,” “Populations,” “Population-based,” etc.).

### Eligibility criteria

Studies were included if they met all of the following conditions: (a) participants were adults (≥18 years) with a confirmed diagnosis of systemic lupus erythematosus (SLE); (b) the primary aim was to examine risk factors for depression among individuals with SLE; (c) depression was assessed using internationally validated rating scales; (d) the design was observational–specifically cross-sectional, cohort, or case–control studies; (e) the full text was published in either English or Chinese; (f) appropriate statistical approaches (e.g., logistic regression) were used to estimate associations; and (g) effect estimates–such as odds ratios (ORs) with 95% confidence intervals (CIs)–were reported directly or could be derived from the provided data.

Exclusion criteria comprised: (a) duplicate or redundant publications; (b) unavailability of the full text despite reasonable retrieval efforts; and (c) insufficient or non-extractable outcome data. Disagreements regarding study eligibility were resolved through consensus between two reviewers, with input from a third reviewer when consensus could not be reached.

### Data extraction process

Two reviewers (LLZ and JJC) independently extracted data using a standardized form to ensure consistency and minimize error. Extracted information included the first author, publication year, study design, country or region, sample size, instruments used to assess depression in SLE patients, and reported risk factors. Any discrepancies were resolved through discussion, with adjudication by a third reviewer (YPS) when consensus could not be reached. The full set of extracted data is summarized in Table 2.

**TABLE 2:** Basic information on included literature (*n* = 26).

References	Country	Study design	Sample size	Assessment tools	Potential risk factor	Quality assessment tools	Quality score (stars)
Chen and Xie (23)	China	Cross-sectional study	305	BDI	k, s, w	AHRQ	10
Yan (24)	China	Cross-sectional study	87	HAMD	m, r	AHRQ	9
Zhou et al. (25)	China	Cross-sectional study	160	SDS	e, k, o	AHRQ	8
Shen et al. (26)	China	Cross-sectional study	93	SDS	c, q	AHRQ	6
Yi (27)	China	Cross-sectional study	402	HADS	s, g, j, t, p	AHRQ	9
Liu et al. (28)	China	Cross-sectional study	70	BDI	b, f, r	AHRQ	9
Li et al. (29)	China	Cross-sectional study	570	Clinical diagnosis	g, r, k	AHRQ	8
Chou (30)	China	Cross-sectional study	140	SDS	o, k, v, w	AHRQ	9
Chen et al. (31)	China	Case-control study	288	SDS	i, n, k, v	NOS	7
Tian et al. (32)	China	Cross-sectional study	202	SDS	s, n, j	AHRQ	7
Julian et al. (33)	USA	Cohort study	663	CES-D	b, c, d, t, s	NOS	8
Huang et al. (34)	USA	Cohort study	1609	Clinical diagnosis	i, r, q, d	NOS	7
Bai et al. (35)	China	Cross-sectional study	176	HAMD	r, l	AHRQ	9
Xie et al. (36)	China	Cross-sectional study	352	HADS	m	AHRQ	10
Abdul-Sattar and Abou El Magd (37)	Egypt	Cross-sectional study	80	CES-D	k, v	AHRQ	8
McCormick et al. (38)	USA	Cross-sectional study	682	CES-D	k, e, c, s	AHRQ	10
Figueiredo-Braga et al. (39)	Portugal	Cohort study	77	HADS	m, w, y	NOS	8
Park et al. (40)	Korea	Cross-sectional study	505	BDI	h, x, c, k, t	AHRQ	10
Parperis et al. (12)	Greece	Cross-sectional study	88	PHQ-9	g, r, s	AHRQ	10
De Souza et al. (41)	Brazil	Cross-sectional study	141	DCS	r, v	AHRQ	10
Chen et al. (42)	China	Cross-sectional study	360	HADS	c, e, l, u, v	AHRQ	9
Hu et al. (43)	China	Case-control study	100	HADS	b, q, r, t, z	NOS	8
Narupan et al. (44)	Thailand	Cross-sectional study	185	PHQ-9	g, i, m, o, v	AHRQ	10
Patterson et al. (45)	USA	Cohort study	431	PHQ-8	d, k, p, s, t	NOS	7
Chawla et al. (46)	USA	Cohort study	763	CES-D	b, c, g, r, k	NOS	8
Hasan et al. (47)	Saudi Arabia	Cross-sectional study	137	BDI	a, f, q, h, t	AHRQ	8

BDI, Beck Depression Inventory; HAMD, Hamilton Depression Scale; SDS, Self-rating Depression Scale; HADS, Hospital Anxiety and Depression Scale; CES-D, Center of Epidemiological Studies Depression Scale; PHQ-9, Patient Health Questionnaire; DCS, Depression Cognition Scale; PHQ-8, Patient Health Questionnaire. a, gender; b, age; c, education attainment; d, race; e, marital status; f, working conditions; g, pain; h, smoking; i, glucocorticoids dose; j, type of health insurance; k, economic conditions; l, anxiety; m, fatigue; n, sleep quality; o, self-image; p, ability to perform activities of daily living; q, course; r, somatic symptoms; s, disease activity; t, comorbidity; u, coping; v, social support; w, relationship satisfaction; x, anticardiolipin antibody positive; y, IL-10, z, Complement C3.

### Quality assessment

Study quality was assessed using instrument-specific criteria. For case–control and cohort studies, the Newcastle–Ottawa Scale (NOS) (21) was applied, with scores of 0–3, 4–6, and 7–9 classified as low, moderate, and high quality, respectively. Cross-sectional studies were evaluated according to the Agency for Healthcare Research and Quality (AHRQ) checklist (22), where scores of 0–3, 4–7, and 8–11 corresponded to low, moderate, and high methodological quality. Any disagreements between the two reviewers during study selection, data extraction, or quality appraisal were resolved through consensus; a third reviewer was consulted when necessary. Detailed quality ratings are provided in Supplementary Tables 1, 2.

### Data analysis

All statistical procedures for this meta-analysis were performed exclusively using STATA software (version 18.0). For each analysis, effect sizes were expressed as odds ratios (ORs) with corresponding 95% confidence intervals (CIs). Given the anticipated clinical and methodological diversity among the included studies, we adopted a conservative approach by applying the random-effects model (DerSimonian-Laird method) across all analyses. Statistical heterogeneity was quantified using the I^2^ statistic with its associated *p*-value; I^2^ values exceeding 50% with *p* < 0.10 were interpreted as indicating substantial heterogeneity. We conducted comprehensive sensitivity analyses by systematically excluding individual studies to evaluate the robustness of our findings and identify potential outliers influencing the overall estimates. Publication bias was assessed through both visual inspection of funnel plots and Egger’s regression test, with statistical significance defined as *p* < 0.05.

## Results

### Literature search and selection

**FIGURE 1:**
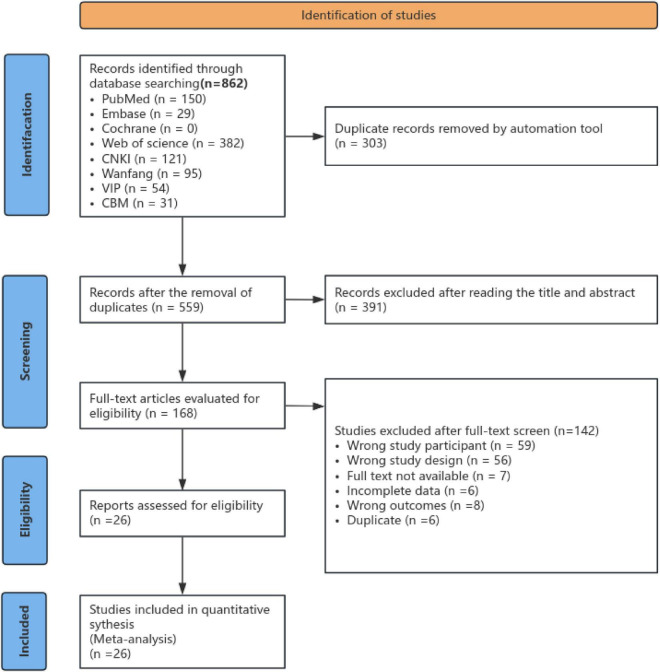
Flow chart of the literature search and study selection in the meta-analysis.

Totally, 862 publications were found, among which 26 studies (12, 23–47) met the inclusion criteria and involved in the qualitative synthesis. The detailed flow chart of the study selection process is shown in Figure 1.

### Characteristics of included studies

There were 26 qualified studies, including 2 case-control studies (31, 43), 5 cohort studies (33, 34, 39, 45, 46), and 19 cross-sectional studies (12, 23–30, 32, 35–38, 40–42, 44, 47), with a total of 8,960 SLE patients involved. Geographically, 14 studies were carried out in China (23–32, 35, 36, 42, 43), 5 in the US (33, 34, 38, 45, 46), 1 in Egypt (37), 1 in Portugal (39), 1 in South Korea (40), 1 in Greece (12), 1 in Brazil (41), 1 in Thailand (44) and 1 in Saudi Arabia (47). Study sample size varied from 70 to 1,609 people. The methodological quality was evaluated with the NOS scale for non-randomized studies and the AHRQ instrument for cross-sectional studies. In the cohort and case-control studies, 4 were rated as 8 stars (high quality) (33, 39, 43, 46) and 3 were rated as 7 stars (31, 34, 45). For cross-sectional studies, 6 scored 10 stars (high quality) (34, 40, 48–51), 6 scored 9 stars (24, 27, 29, 30, 35, 42), 4 scored 8 stars (25, 28, 37, 47), 1 scored 7 stars (32), and 1 scored 6 stars (26), which shows that the overall quality of the included literature is good. Full quality assessment result is shown in Table 2, see Supplementary Tables 3, 4 for detailed information. Nine different instruments were used to evaluate depression across the studies. The BDI was used in 4 studies (23, 28, 40, 47), the CES-D was used in 4 studies (33, 37, 38, 46), and the PHQ-9 was used in 2 studies (12, 44). HADS was used in 4 studies (12, 25, 34, 37), and SDS was used in 5 studies (25, 26, 30–32). Other tools include: DCS [depression clinical scale; 1 study (41)]; clinical diagnosis [2 studies (29, 34)]; HAMD [Hamilton Depression Rating Scale; 2 studies (24, 35)]; and PHQ-8 [Patient Health Questionnaire-8; 1 study (45)] (Table 2).

### Sensitivity analysis

#### Meta-analysis results

##### Depression prevalence and subgroup analyses in SLE patients

This study initially identified 26 cross-sectional studies. However, two did not report sufficient data to calculate depression prevalence and were therefore excluded, resulting in a final sample of 24 studies (12, 23–32, 35–47) involving 5,794 patients with systemic lupus erythematosus (SLE)–including 2,887 from China (12 studies) and 2,907 from other regions (12 studies). All 24 included studies reported depression prevalence. To stabilize variance, we applied the Freeman–Tukey double arcsine transformation and estimated the pooled prevalence using a DerSimonian–Laird random-effects model.

The overall pooled prevalence of depression among SLE patients was 39.4% (95% CI: 32.4–46.7). Substantial between-study heterogeneity was observed (*P* < 0.001; I^2^ = 96.9%), justifying the use of a random-effects model.

Subgroup analysis by geographic region revealed a significantly higher depression burden in Chinese cohorts compared with non-Chinese populations (*P* = 0.046):

China: pooled prevalence = 53.2% (95% CI: 38.3–67.6; I^2^ = 97.9%; *n* = 2,887, 12 studies)

Non-China: pooled prevalence = 27.8% (95% CI: 22.0–34.2; I^2^ = 91.6%; *n* = 2,907, 12 studies)

To explore sources of residual heterogeneity within subgroups (China: I^2^ = 97.9%; non-China: I^2^ = 91.6%), we conducted meta-regression. Results indicated that: a 1% increase in the proportion of female participants was associated with a significant rise in depression prevalence (β = 0.055, *P* = 0.026);

Each additional year in mean age corresponded to a significant decline in prevalence (β = −0.048, *P* = 0.004);

The type of depression assessment tool (HADS vs. BDI, PHQ-9, or CES-D) showed no significant effect (β = −0.126, *P* = 0.433).

**FIGURE 2:**
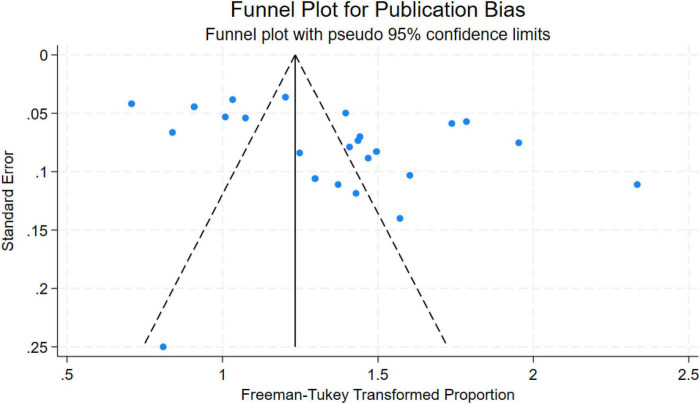
Funnel plot for publication bias in depression rates among SLE patients.

Egger’s regression test detected significant small-study effects or publication bias (intercept = 28.94, *P* < 0.001), and funnel plot asymmetry (see Figure 2) suggested that smaller studies tended to report higher prevalence estimates. Nevertheless, leave-one-out sensitivity analysis confirmed the robustness of the main estimate: exclusion of any single study yielded pooled prevalences ranging from 37.5% to 40.9%, with no meaningful shift in direction or magnitude.

### Risk factors for depression in patients with SLE

Among the 26 included studies, we identified over 20 potential correlates of depression in SLE patients across sociodemographic, socioeconomic, clinical, and patient-reported domains. Eleven factors met criteria for meta-analysis. As shown in Table 3, younger age, lower education, economic hardship, unemployment, smoking, higher disease activity, high-dose glucocorticoids, comorbidity, and greater fatigue were significantly associated with increased depression risk (all *P* < 0.05). Disease duration was not associated (OR = 0.90, *P* = 0.40), and low social support did not reach significance (*P* = 0.064). Remaining variables–including ethnicity, marital status, pain, sleep quality, body image, TCM-specific symptoms (e.g., menstrual irregularity), organ involvement, laboratory markers (e.g., low C3), and insurance type–were reported in ≤2 studies or measured inconsistently (e.g., “family burden” vs. “family dysfunction”) and were therefore summarized narratively (see Supplementary Table 5).

**TABLE 3:** Meta-analysis results of risk factors of depression in patients with SLE.

Risk factors	Number of articles included	*I*^2^ (%)	*P*	OR	95% CI
Age (younger vs. older)	3 (29, 33, 12)	10.9	<0.001**	1.97	1.41, 2.76
(per 1-year increase)	2 (43, 46)	94.7	0.645	1.03	0.91, 1.16
Academic qualifications (lower vs. higher)	3 (33, 40, 42)	1.2	<0.001**	2.21	1.45, 3.36
Economic hardship (yes vs. no)	6 (30, 31, 37, 38, 45, 46)	28.8	<0.001**	6.05	3.64, 10.07
Unemployment (yes vs. no)	3 (29, 12, 47)	21.6	0.003*	3.06	1.48, 6.32
Social support (low vs. high)	3 (31, 42, 44)	44.9	0.06	7.06	0.89, 56.00
Smoking (yes vs. no)	2 (38, 46)	0	0.004*	3.17	1.44, 6.99
Disease duration (longer vs. shorter)	4 (28, 34, 43, 47)	80.4	0.404	0.90	0.69, 1.16
Disease activity (higher vs. lower)	4 (27, 32, 33, 38)	0	0.02*	3.15	2.95, 3.37
Glucocorticoids dose (high-dose vs. low)	4 (28, 31, 34, 44)	0	<0.001**	7.72	4.62, 12.90
Comorbidity (presence vs. absent)	2 (27, 47)	0	<0.001**	2.20	1.45, 3.34
Fatigue (per unit increase)	3 (36, 39, 44)	29.4	0.002**	1.23	1.08, 1.40

*p < 0.05,

**p < 0.01.

#### Age

**FIGURE 3:**
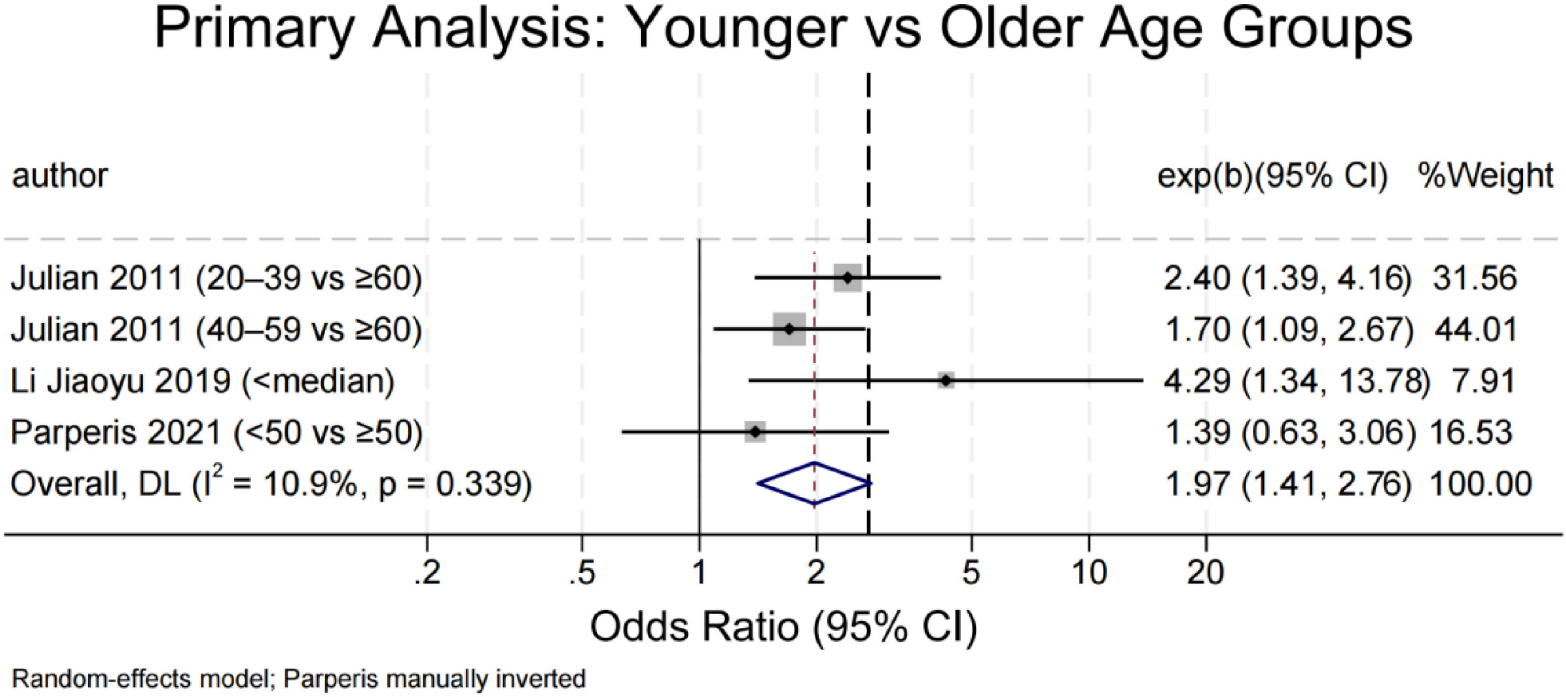
Forest plot of depression risk in SLE patients by age group (younger vs. older).

**FIGURE 4:**
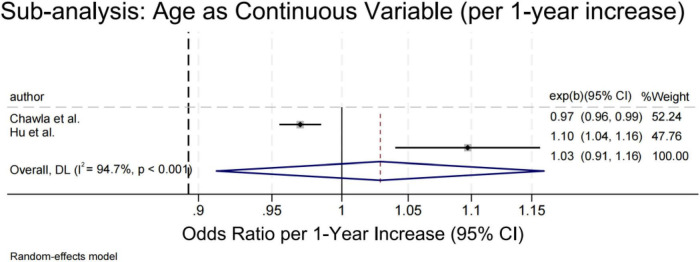
Forest plot of depression risk in SLE patients by age (continuous variable).

Four studies compared depression risk between younger and older adults using categorical age thresholds (Figure 3) (<50 years or study-specific medians) (12, 29, 33), while two modeled age continuously (43, 46). Younger age was consistently associated with higher odds of depression (OR = 1.97; 95% CI: 1.41–2.76; *P* < 0.001), with low heterogeneity (I^2^ = 10.9%). Sensitivity analyses remained significant upon exclusion of individual studies, and Egger’s test showed no evidence of bias (*P* = 0.510). In contrast, continuous age models (Figure 4) yielded conflicting results–Chawla et al. (46) reported a protective effect (OR = 0.97), whereas Hu et al. (43) found increased risk (OR = 1.10). The pooled estimate was non-significant (OR = 1.03; *P* = 0.645) with high heterogeneity (I^2^ = 94.7%), indicating that the age–depression relationship is context-dependent and sensitive to analytical approach.

#### Academic qualifications

Lower education was linked to significantly elevated depression risk (OR = 2.21; 95% CI: 1.45–3.36; *P* < 0.001; I^2^ = 1.2%) across three studies (33, 40, 42). No publication bias was evident (Egger’s *P* = 0.112), and sensitivity analyses confirmed stability.

#### Economic hardship

Defined primarily by low income, economic hardship was strongly associated with depression (OR = 6.05; 95% CI: 3.64–10.07; *P* < 0.001; I^2^ = 28.8%) across six studies (30, 31, 37, 38, 45, 46). The effect was consistent in U.S. (OR = 7.42) and Asian cohorts (OR = 7.14; *P* = 0.962 for subgroup difference), underscoring its cross-cultural relevance. No publication bias was detected (Begg’s *P* = 0.260; Egger’s *P* = 0.385).

#### Unemployment

Three studies (12, 29, 47) reported a significant association (OR = 2.46; 95% CI: 1.51–4.02; I^2^ = 6.2%), with a stronger effect in Asian populations (OR = 3.06) than in the single European study (OR = 1.85). Results were robust to sensitivity testing.

Meta-analysis of three observational studies (*n* = 3) indicated that unemployment was significantly associated with a higher risk of depression among SLE patients (OR = 2.46, 95% CI: 1.51–4.02; I^2^ = 6.2%). Subgroup analysis suggested a stronger association in Asian populations (OR = 3.06), while the single European study showed a non-significant trend (OR = 1.85). Sensitivity analysis confirmed robustness, and Egger’s test did not suggest significant publication bias (*p* = 0.324).

#### Social support

Three studies (31, 42, 44) examined low social support and depression. The pooled OR was 7.06 (95% CI: 0.89–56.00; *P* = 0.064), with moderate heterogeneity (I^2^ = 44.9%). The estimate was highly sensitive to individual studies–particularly Chen et al. (31) –and became non-significant upon its exclusion. Given the small number of studies and methodological differences in how social support was measured, this association should be interpreted cautiously. Bias tests (Egger’s *P* = 0.144; Begg’s *P* = 0.602) lacked power but showed no clear asymmetry.

#### Smoking

Only two studies (40, 47) were available for meta-analysis on the link between smoking and disease risk. A random-effects model yielded a pooled odds ratio of 3.17 (95% CI: 1.44–6.99; *P* = 0.004), suggesting that smokers may face a higher risk of developing the disease compared to non-smokers. Between-study heterogeneity was negligible (I^2^ = 0%; *P* = 0.356), but leave-one-out analysis revealed high influence from Park et al. (40). This finding is considered exploratory and requires replication.

#### Disease duration

Four observational studies (28, 34, 43, 47) examined the association between disease duration and depression risk in patients with SLE, yet their findings pointed in opposing directions. Two (28, 34) suggested a protective effect of longer duration (pooled RR = 0.77), while two (43, 47) indicated increased risk (pooled OR = 1.69). Meta-analysis of all four showed no overall association (pooled OR/RR = 0.90; *P* = 0.40; I^2^ = 80.4%), suggesting a non-linear or population-specific relationship.

#### Disease activity

Four studies (27, 32, 33, 38) assessed SLE disease activity and depression. Excluding Tian et al. (38), the main analysis (*n* = 3) showed a strong positive association (OR = 3.15, 95% CI: 2.95–3.37; I^2^ = 0%). Including Tian et al. (38) (OR = 1.51, 95% CI: 1.23–2.25) reduced the overall estimate to OR = 2.80 (95% CI: 2.24–3.50), still highly significant. Subgroup analysis by publication year showed consistent directionality: earlier studies (2011–2018) yielded OR = 3.15, while the 2023 study reported a weaker but positive effect. No publication bias was detected (Begg’s *P* = 1.0; Egger’s *P* = 0.45). Sensitivity analysis confirmed that the core finding was driven by the three earlier, highly homogeneous studies.

#### Glucocorticoid dose

Four studies (28, 31, 34, 44) examined high-dose glucocorticoid use and depression. Given the low prevalence of depression in SLE cohorts, relative risks (RRs) from two studies (28, 34) were conservatively treated as equivalent to odds ratios (ORs). The pooled OR was 7.72 (95% CI: 4.62–12.90; *P* < 0.001), with no heterogeneity (I^2^ = 0%). Stratification by effect measure type showed consistent direction: RR-based studies (OR = 7.01) and OR-based studies (OR = 15.82, though imprecise). Between-subgroup heterogeneity was non-significant (*P* = 0.31). Egger’s test suggested possible small-study effects (*P* = 0.04), largely driven by Narupan et al. (44) (wide CI: 1.76–18.8). Sensitivity analysis confirmed robustness: pooled ORs ranged from 6.5 to 10.2 upon exclusion of any single study.

#### Comorbidity

Two studies (27, 47) reported on comorbidity status and depression. The pooled OR was 2.20 (95% CI: 1.45–3.34; *P* < 0.001), with no heterogeneity (I^2^ = 0%). Leave-one-out analysis yielded nearly identical estimates (OR = 2.14 or 2.30), confirming robustness.

#### Fatigue

Three cross-sectional studies (36, 39, 44) from China, Portugal, and Thailand examined fatigue and depression. Because depression instruments differed, only two studies using the HADS-D subscale (36, 39) were pooled. Each unit increase in fatigue was associated with 20% higher odds of depressive symptoms (OR = 1.20; 95% CI: 1.10–1.32; I^2^ = 0%). The third study (44), using PHQ-9, reported a larger effect (OR = 2.36) but was excluded from the main analysis due to measurement incompatibility. Sensitivity analysis confirmed stability, and Egger’s test showed no small-study bias (*P* = 0.33).

## Discussion

This systematic review and meta-analysis of 26 studies involving 8,960 patients with SLE revealed a pooled prevalence of depression of 39.4%, indicating that nearly two in five individuals with SLE experience clinically relevant depressive symptoms. Notably, substantial geographic variation was observed: over half of Chinese patients (53.2%) screened positive for depression, compared with only 27.8% in non-Chinese cohorts. This marked disparity suggests that geographic context–potentially reflecting differences in healthcare access, cultural attitudes toward mental health, or diagnostic practices–plays a critical role in shaping depression risk among SLE patients.

We further identified a consistent set of sociodemographic, clinical, and psychosocial correlates–including younger age, female sex, lower academic qualifications, economic hardship, unemployment, high disease activity, and glucocorticoid dose. Collectively, these findings suggest that depression in SLE is not merely a reactive emotional response to chronic illness but a multifactorial syndrome shaped by intertwined biological, social, and cultural forces.

### Overall prevalence and geographic disparities

Our estimated prevalence (39.4%) is somewhat higher than those reported in earlier meta-analyses (typically 24%–39%) (7), possibly reflecting broader screening practices, heightened clinical awareness, or rising societal stressors in recent years. The markedly elevated burden among Chinese patients–nearly double that of other regions–is particularly concerning. While methodological differences (e.g., recruitment from tertiary referral centers where disease severity may be greater) may partially account for this gap, deeper contextual factors are likely at play. In many Asian societies, including China, mental health conditions remain heavily stigmatized, often leading individuals to delay seeking help until symptoms become severe (48). Our meta-regression analysis suggests that demographic features common in Chinese cohorts–namely, a higher proportion of women and younger average age–partially mediate this disparity, consistent with established epidemiological patterns showing elevated depression risk in women and young adults (49, 50).

### Socioeconomic disadvantage as a central driver

Economic hardship emerged as the strongest correlate of depression (OR ≈ 6.05), with consistent effects across regions. This aligns with the social determinants of health framework: financial strain restricts access to medications and specialist care while simultaneously fueling chronic stress, sleep disruption, and social isolation–all of which have neurobiological links to mood dysregulation (51). Similarly, low education (OR = 2.21) and unemployment (OR = 2.46) independently increased depression risk, underscoring how structural inequities shape mental health outcomes in SLE.

Loss of employment not only reduces income but also erodes social identity and daily routine (52, 53); conversely, work participation can divert attention from illness, foster positive affect, and strengthen support networks through workplace interactions (54). These insights support integrating socioeconomic screening into routine rheumatology visits and advocate for policy-level interventions–such as income support, vocational rehabilitation, and reintegration programs–for patients with chronic autoimmune diseases.

### Biological contributors: inflammation and medication effects

Clinically, both high disease activity and glucocorticoid therapy were among the most potent predictors of depression. The robust association with disease activity supports the “inflammatory-depression” hypothesis: pro-inflammatory cytokines (e.g., IL-6, TNF-α) can cross the blood–brain barrier, disrupt monoamine metabolism, and dysregulate the hypothalamic–pituitary–adrenal axis, thereby directly promoting depressive symptoms (55). Meanwhile, high-dose glucocorticoid use showed an exceptionally strong link with depression (OR = 7.72), corroborating well-documented neuropsychiatric side effects ranging from mood lability and insomnia to steroid-induced psychosis (34, 56–58). Clinicians should therefore consider depression in active SLE not just as a comorbidity but as a potential manifestation of uncontrolled inflammation or iatrogenic drug effects.

### Behavioral and psychosocial factors: complexity with intervention potential

Smoking, though assessed in fewer studies (OR = 3.17) (59), presents a modifiable risk factor with plausible biological mechanisms–namely, oxidative stress and neuroinflammation that heighten vulnerability to mood disorders (60, 61). Integrating smoking cessation support into routine SLE care could thus reduce psychiatric comorbidity.

Fatigue–one of the most disabling SLE symptoms–was strongly linked to depression. The FATILUP study (62) confirmed their tight interrelationship, and Da Costa et al. (63) identified depression as the strongest predictor of mental fatigue. Given the profound impact of fatigue on quality of life, evidence-based strategies such as supervised aerobic exercise have shown promise in alleviating both physical and emotional burden (64).

## Limitations

This review is limited by the cross-sectional nature of included studies, preventing causal inference. High heterogeneity (I^2^ > 75%) persisted despite subgroup analyses, likely due to unmeasured cultural or clinical confounders. Publication bias was detected but did not alter core conclusions in sensitivity analyses. Most studies originated from China, potentially limiting generalizability. Crucially, inconsistent measurement and sparse reporting of psychosocial factors (e.g., pain, family functioning) hindered meta-analysis, underscoring the need for a standardized Core Outcome Set in SLE research.

## Conclusion

The risk of depression in patients with SLE arises from a complex interplay of biological, psychological, and social factors. In clinical practice, beyond monitoring disease activity and pharmacological management, healthcare providers should routinely and systematically assess patients’ socioeconomic circumstances–including financial strain, educational attainment, employment status, availability of social support, and distress related to body image. Only through an integrated approach that combines medical, psychological, and social interventions–centered on a holistic model of person-centered care–can the mental health outcomes of this vulnerable population be meaningfully improved.

## Data Availability

The original contributions presented in this study are included in this article/Supplementary material, further inquiries can be directed to the corresponding authors.
