# Supplementary material for: Risk factors for depression in systemic lupus erythematosus: a systematic review and meta-analysis
Source: Front Med (Lausanne). 2026 Feb 19;13:1751870. doi: 10.3389/fmed.2026.1751870 (PMC12960599; doi:10.3389/fmed.2026.1751870)
Supplement: Supplementary file 1 [file Table_1.docx]

# Supplementary Table S1: Newcastle-Ottawa Quality Assessment Scale

| **Cohort studies** |
| --- |
| *Selection* |
| 1. Representativeness of the exposed cohort 2. Truly representative of the average _______________ (describe) in the community ­­ 3. Somewhat representative of the average ______________ in the community ­ 4. Selected group of users eg, nurses, volunteers 5. No description of the derivation of the cohort |
| 1. Selection of the non-exposed cohort 2. Drawn from the same community as the exposed cohort ­ 3. Drawn from a different source 4. No description of the derivation of the non-exposed cohort |
| 1. Ascertainment of exposure 2. Secure record (eg, surgical records) ­ 3. Structured interview ­ 4. Written self-report 5. No description |
| 1. Demonstration that outcome of interest was not present at start of study 2. Yes ­ 3. No |
| *Comparability* |
| 1. Comparability of cohorts on the basis of the design or analysis 2. Study controls for _____________ (select the most important factor) ­ 3. Study controls for any additional factor ­ (this criterion could be modified to indicate specific control for a second important factor) |
| *Outcome* |
| 1. Assessment of outcome 2. Independent blind assessment ­ 3. Record linkage ­ 4. Self-report 5. No description |
| 1. Was follow up long enough for outcomes to occur 2. Yes (select an adequate follow-up period for outcome of interest) ­ 3. No |
| 1. Adequacy of follow up of cohorts 2. Complete follow up – all subjects accounted for ­ 3. Subjects lost to follow up unlikely to introduce bias – small number lost – > ____ % (select an adequate %) follow up, or description provided of those lost) ­ 4. Follow-up rate < ____% (select an adequate %) and no description of those lost 5. No statement |

Note: A study can be awarded a maximum of one star for each numbered item within the Selection and Outcome categories. A maximum of two stars can be given for Comparability

| **Case-control studies** |
| --- |
| *Selection* |
| 1. Is the case definition adequate? 2. Yes, with independent validation ­ 3. Yes, e.g. record linkage or base on self-reports 4. No description |
| 1. Representativeness of the cases 2. Consecutive or obviously representative series of cases ­ 3. Potential for selection biases or not stated |
| 1. Selection of Controls 2. Community controls ­ 3. Hospital controls 4. No description |
| 1. Definition of Controls 2. No history of disease (endpoint) ­ 3. No description of source |
| *Comparability* |
| 1. Comparability of case and controls on the basis of the design or analysis 2. Study controls for _____________ (select the most important factor) ­ 3. Study controls for any additional factor ­ (this criterion could be modified to indicate specific control for a second important factor. ) |
| *Exposure* |
| 1. Assessment of exposure 2. Secure record (e.g. surgical records) ­ 3. Structured interview where blind to case/control status ­ 4. Interview not blinded to case/control status 5. Written self-report or medical record only 6. No description |
| 1. Same method of ascertainment for case and controls 2. Yes ­ 3. No |
| 1. Non-Response rate 2. same rate for both groups ­ 3. Non respondents described 4. Rate different and no designation |

Note: A study can be awarded a maximum of one star for each numbered item within the Selection and Exposure categories. A maximum of two stars can be given for Comparability.
