# Supplementary material for: Risk factors for depression in systemic lupus erythematosus: a systematic review and meta-analysis
Source: Front Med (Lausanne). 2026 Feb 19;13:1751870. doi: 10.3389/fmed.2026.1751870 (PMC12960599; doi:10.3389/fmed.2026.1751870)
Supplement: Supplementary file 2 [file Table_2.docx]

**Supplementary Table S2: Agency for Healthcare Research and Quality**

| Evaluation item | Yes | No | Unclear |
| --- | --- | --- | --- |
| 1.Define the source of information (survey, record review). |  |  |  |
| 2.List inclusion and exclusion criteria for exposed and unexposed subjects (cases and controls) or refer to previous publications. |  |  |  |
| 3.Indicate time period used for identifying patients. |  |  |  |
| 4.Indicate whether or not subjects were consecutive if not population-based. |  |  |  |
| 5.Indicate if evaluators of subjective components of study were masked to other aspects of the participants. |  |  |  |
| 6.Describe any assessments undertaken for quality assurance purpose (e.g., test/retest of primary outcome measurements). |  |  |  |
| 7.Explain any patient exclusions from analysis. |  |  |  |
| 8.Describe how confounding was assessed and/or controlled. |  |  |  |
| 9.If applicable, explain how missing data were handled in the analysis. |  |  |  |
| 10.Summarize patient response rates and completeness of data collection. |  |  |  |
| 11.Clarify what follow-up, if any, was expected and the percentage of patients for which incomplete data or follow-up was obtained. |  |  |  |
