# Supplementary material for: Risk factors for depression in systemic lupus erythematosus: a systematic review and meta-analysis
Source: Front Med (Lausanne). 2026 Feb 19;13:1751870. doi: 10.3389/fmed.2026.1751870 (PMC12960599; doi:10.3389/fmed.2026.1751870)
Supplement: Supplementary file 3 [file Table_3.docx]

# Supplementary Table S3: Quality assessment of NOS

| Author. year of publication | Selection | Comparability | Exposure/Outcome | Total score |
| --- | --- | --- | --- | --- |
| Cohort studies |  |  |  |  |
| Julian et al. 2011 (33) | *** | ** | *** | ******** |
| Huang et al. 2014 (34) | *** | ** | ** | ******* |
| Figueiredo-Braga et al. 2018 (39) | **** | ** | ** | ******** |
| Patterson et al. 2022 (46) | *** | ** | ** | ******* |
| Chawla et al. 2022 (47) | *** | ** | *** | ******** |
| Case-control studies |  |  |  |  |
| Chen Ru Meng et al. 2022 (31) | *** | ** | ** | ******* |
| Hu et al. 2022 (44) | **** | ** | ** | ******** |
