# Supplementary material for: Risk factors for depression in systemic lupus erythematosus: a systematic review and meta-analysis
Source: Front Med (Lausanne). 2026 Feb 19;13:1751870. doi: 10.3389/fmed.2026.1751870 (PMC12960599; doi:10.3389/fmed.2026.1751870)
Supplement: Supplementary file 4 [file Table_4.docx]

**Supplementary Table S4: Quality assessment of AHRQ**

| Author. year of publication | 1 | 2 | 3 | 4 | 5 | 6 | 7 | 8 | 9 | 10 | 11 | Total score |
| --- | --- | --- | --- | --- | --- | --- | --- | --- | --- | --- | --- | --- |
| Chen Pei Ling et al. 2013 (23) | Y | Y | Y | Y | Y | Y | Y | Y | U | Y | Y | 10 |
| Yan Jing Yao. 2013 (24) | Y | Y | U | Y | Y | Y | Y | Y | U | Y | Y | 9 |
| Zhou Feng Yan et al. 2016 (25) | Y | Y | Y | Y | Y | Y | N | Y | U | Y | N | 8 |
| Shen Hui Zhu et al. 2018(26) | Y | N | N | Y | Y | Y | N | Y | U | N | Y | 6 |
| Yi Pei Wen. 2018 (27) | Y | Y | N | Y | Y | Y | Y | Y | U | Y | Y | 9 |
| Liu Shu Yi et al. 2019 (28) | Y | Y | N | Y | Y | Y | N | Y | U | Y | Y | 8 |
| Li Jiao Yu et al. 2019 (29) | Y | Y | N | Y | Y | Y | N | Y | Y | Y | Y | 9 |
| Qiu Ya Yun. 2020 (30) | Y | Y | Y | Y | Y | Y | Y | Y | U | Y | N | 9 |
| Tian Dan Dan et al. 2023 (32) | Y | Y | N | Y | Y | Y | U | Y | U | Y | N | 7 |
| Bai et al. 2016 (35) | Y | Y | Y | Y | Y | Y | N | Y | U | Y | Y | 9 |
| Xie et al. 2016 (36) | Y | Y | Y | Y | Y | Y | Y | Y | U | Y | Y | 10 |
| Abdul-Sattar et al. 2017 (37) | Y | Y | N | Y | Y | Y | N | Y | U | Y | Y | 8 |
| McCormick et al. 2017(38) | Y | Y | Y | Y | Y | Y | Y | Y | U | Y | Y | 10 |
| Park et al. 2018 (40) | Y | Y | Y | Y | Y | Y | N | Y | Y | Y | Y | 10 |
| Parperis et al. 2021 (41) | Y | Y | Y | Y | Y | Y | Y | Y | U | Y | Y | 10 |
| Souza et al. 2021 (42) | Y | Y | Y | Y | Y | Y | Y | Y | U | Y | Y | 10 |
| Chen et al. 2022 (43) | Y | Y | Y | Y | Y | Y | Y | Y | U | Y | U | 9 |
| Narupan et al. 2022 (45) | Y | Y | Y | Y | Y | Y | Y | Y | U | Y | Y | 10 |
| Hasan et al. 2024 (48) | Y | Y | N | Y | Y | N | Y | Y | U | Y | Y | 8 |
